# RETRACTED: Farag, R.K.; Mohamed, R.R. Synthesis and Characterization of Carboxymethyl Chitosan Nanogels for Swelling Studies and Antimicrobial Activity. *Molecules* 2013, *18*, 190–203

**DOI:** 10.3390/molecules31071133

**Published:** 2026-03-30

**Authors:** Reem K. Farag, Riham R. Mohamed

**Affiliations:** 1 Egyptian Petroleum Research Institute (EPRI), Nasr City, Cairo 11727, Egypt; reem_kamal.kamel2009@yahoo.com; 2 Department of Chemistry, Faculty of Science, Cairo University, Giza 12613, Egypt

The journal retracts the article titled “Synthesis and Characterization of Carboxymethyl Chitosan Nanogels for Swelling Studies and Antimicrobial Activity” [1], cited above.

Following publication, concerns were brought to the attention of the Editorial Office regarding the integrity of a number of images presented in this article [1].

In accordance with standard journal procedures, the Editorial Office and Editorial Board conducted an investigation that identified indications of inappropriate editing and duplication within figures presented in this article, as well as between this article and an earlier publication [2], produced by a similar authorship group. Although the authors cooperated with the investigation, the explanations and supporting materials provided were not deemed sufficient by the Editorial Board to resolve the identified concerns. As a consequence, the Editorial Board has lost confidence in the reliability of the reported findings and has decided to retract this publication in accordance with MDPI’s retraction policy.

This retraction was approved by the Editor-in-Chief of the journal *Molecules*.

The authors have been informed of this retraction and have indicated their disagreement with this decision.
